# Net water uptake within the ischemic penumbra predicts the presence of the midline shift in patients with acute ischemic stroke

**DOI:** 10.3389/fneur.2023.1246775

**Published:** 2023-09-29

**Authors:** Cuiping Chen, Jianhong Yang, Qing Han, Yuefei Wu, Jichuan Li, Tianqi Xu, Jie Sun, Xiang Gao, Yi Huang, Mark W. Parsons, Longting Lin

**Affiliations:** ^1^Department of Neurology, The First Affiliated Hospital of Ningbo University, Ningbo, Zhejiang, China; ^2^Department of Neurosurgery, The First Affiliated Hospital of Ningbo University, Ningbo, Zhejiang, China; ^3^Department of Neurology, Liverpool Hospital, Sydney, NSW, Australia; ^4^Sydney Brain Center, University of New South Wales, Sydney, NSW, Australia

**Keywords:** stroke, anterior circulation infarction, brain edema, midline shift, net water uptake

## Abstract

**Objective:**

The study aimed to explore the association between midline shift (MLS) and net water uptake (NWU) within the ischemic penumbra in acute ischemic stroke patients.

**Methods:**

This was a retrospective cohort study that examined patients with anterior circulation stroke. Net water uptake within the acute ischemic core and penumbra was calculated using data from admission multimodal CT scans. The primary outcome was severe cerebral edema measured by the presence of MLS on 24 to 48 h follow-up CT scans. The presence of a significant MLS was defined by a deviation of the septum pellucidum from the midline on follow-up CT scans of at least 3 mm or greater due to the mass effect of ischemic edema. The net water uptake was compared between patients with and without MLS, followed by logistic regression analyses and receiver operating characteristics (ROCs) to assess the predictive power of net water uptake in MLS.

**Results:**

A total of 133 patients were analyzed: 50 patients (37.6%) with MLS and 83 patients (62.4%) without. Compared to patients without MLS, patients with MLS had higher net water uptake within the core [6.8 (3.2–10.4) vs. 4.9 (2.2–8.1), *P* = 0.048] and higher net water uptake within the ischemic penumbra [2.9 (1.8–4.3) vs. 0.2 (−2.5–2.7), *P < * 0.001]. Penumbral net water uptake had higher predictive performance than net water uptake of the core in MLS [area under the curve: 0.708 vs. 0.603, *p* < 0.001]. Moreover, the penumbral net water uptake predicted MLS in the multivariate regression model, adjusting for age, sex, admission National Institutes of Health Stroke Scale (NIHSS), diabetes mellitus, atrial fibrillation, ischemic core volume, and poor collateral vessel status (OR = 1.165; 95% CI = 1.002–1.356; *P* = 0.047). No significant prediction was found for the net water uptake of the core in the multivariate regression model.

**Conclusion:**

Net water uptake measured acutely within the ischemic penumbra could predict severe cerebral edema at 24–48 h.

## Introduction

Stroke is one of the leading causes of death and disability worldwide (1). Cerebral edema after acute ischemic stroke (AIS) often leads to poor clinical outcome and increases mortality (1, 2). Midline shift (MLS) describes the horizontal displacement degree of the septum pellucidum on a CT scan, and it has long been established as a radiologic feature of severe brain edema (3–6). However, MLS often manifests after 24 h of stroke onset, frequently leading to delayed identification (7–9). At the delayed stage, the treatment effect of osmotherapy or surgical decompressive craniectomy would be compromised, and it might be difficult to prevent the rapid progression of edema, which might eventually lead to cerebral herniation or demise in some malignant cases (10–12). Therefore, the detection of early indicators of severe brain edema is essential, as it can allow clinicians to promptly initiate preventive therapeutic strategies.

Net water uptake (NWU) has been reported to help predict brain edema in the hyperacute stage of acute ischemic stroke. It quantifies the degree of hypoattenuation on computed tomography (CT) scans (13, 14). Prior studies have reported that NWU within the ischemic core is a promising early biomarker of malignant cerebral edema (8, 15, 16). The majority of NWU research focuses on the ischemic core, whereas little research has been conducted on the ischemic penumbra. Ischemic penumbra refers to mild-moderate hypoperfusion brain tissues that are salvageable with prompt blood reperfusion (17, 18). It has been the target of reperfusion treatment, either endovascular thrombectomy or intravenous thrombolysis. However, recent studies have shown that reperfusion of the penumbral tissues might lead to reperfusion injury, including edema and bleeding (19). In a recent study published by our group, we showed that NWU within the penumbra predicted symptomatic intracranial hemorrhages (20). This study aimed to explore whether NWU in the penumbral region can predict severe brain edema measured by MLS.

## Methods

### Study design

This was a retrospective cohort study. Patients were dichotomized into the following two groups: (1) MLS group, including patients with severe brain edema defined by middle line shift (MLS), and (2) non-MLS group, including patients with no MLS. The MLS and non-MLS groups were compared in terms of baseline net water uptake in the region of the ischemic penumbra and core, respectively.

### Patients

This study retrospectively screened patients who were diagnosed with their first-ever acute ischemic stroke (within 24 h of symptom onset) in the First Affiliated Hospital of Ningbo University from July 2017 to September 2019. This study selected patients with the following inclusion criteria: (1) acute vessel occlusion of the anterior circulation; (2) multimodal CT imaging completed within 24 h of admission, including non-contrast CT (NCCT) scans, CT angiography (CTA), and CT perfusion (CTP); and (3) follow-up CT scans completed 24 to 48 h after hospitalization. The key exclusion criterion was abnormal density alteration on admission NCCT caused by pre-existing infarction or hemorrhage. This study was approved by the ethics committee of the First Affiliated Hospital of Ningbo University, and all patients signed the informed consent form for the collected data before being included in the study.

### Clinical data

Clinical data included age, sex, severity of ischemic stroke on admission measured by the National Institutes of Health Stroke Scale (NIHSS), and medical history.

### Image data

The patients received a comprehensive one-stop stroke imaging protocol by multimodal CT scans at admission with NCCT, CTA, and CTP on a 320-slice scanner (Toshiba Aquilion ONE, Canon Medical Imaging, Tokyo, Japan). Collateral status was assessed on the CTA with ASITN/SIR collateral scores ranging from 0 to 4, and poor collateral status was defined as scores ≤2.

The CTP image was spatially segmented into 320 axial sections with 0.5 mm thickness per layer and temporally divided into 19 time points beginning at 4 s after non-ionic iodinated contrast injection into an antecubital vein. The CTP data were processed using commercial software (MI-Star, Apollo Medical Imaging Technology, Melbourne, VIC, Australia). The ischemic core and ischemic penumbra were recognized by CTP and were defined as follows: ischemic core was delay time (DT) >3 s and cerebral blood flow (CBF) <30%, while ischemic penumbra means DT >3 s and CBF >30% according to the corresponding threshold (21).

### Net water uptake

**Figure 1:**
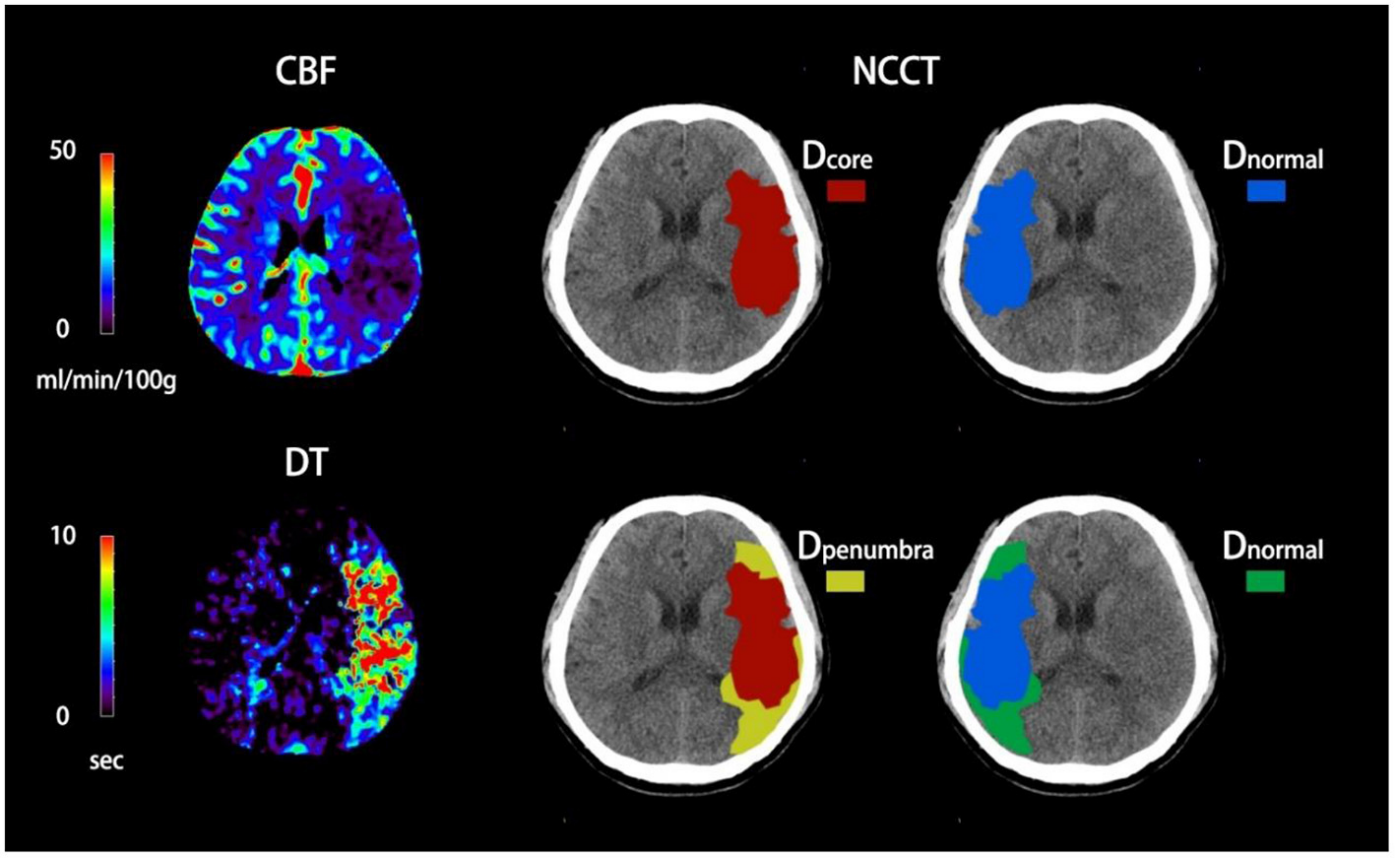
Quantification of NWU-core and NWU-penumbra in admission non-contrast computed tomography (NCCT). The ischemic core and penumbra were identified by cerebral blood flow (CBF) and delay time (DT) maps based on computed tomography perfusion (CTP) with the setting of different thresholds. The mean density of the ischemic core, ischemic penumbra, and normal tissue derived from the mirrored contralateral were then calculated.

Figure 1 shows how net water uptake was measured. First, the regions of interest (ROIs) of the ischemic core and penumbra were manually drawn according to CTP, and then mirrored contralateral ROIs were generated automatically and defined as normal tissue. Both ROIs were superimposed on NCCT and were sampled with CT density between 20 and 80 Hounsfield units (HU) to exclude voxels, including CSF or calcification.

NWU-core measured an elevated volume of water uptake per unit volume of ischemic core, while NWU-penumbra represented a higher volume of water uptake per unit volume of ischemic penumbra. The correlative equations were as follows (22, 23):


$$\begin{array}{r} NWUcore=\frac{\text{Vwater}}{\text{Vcore}}=\left(\frac{\text{1}-\text{Dcore}}{\text{Dnormal}}\right)\times \text{100}\%\;\\ NWUpenumbra=\frac{\text{Vwater}}{\text{Vpemumbra}}=\left(\frac{\text{1}-\text{Dpenumbra}}{\text{Dnormal}}\right)\times \text{100}\%\; \end{array}$$

### Outcome

The primary imaging endpoint of this study was the presence of significant MLS. MLS was measured as the maximum horizontal deviation of the line between the anterior and posterior attachment points of the falx cerebri on follow-up CT scans. The presence of significant MLS was defined by a deviation of at least 3 mm or greater due to the mass effect of ischemic edema (24). Two double-blinded clinicians reviewed the CT images to confirm the presence of significant MLS. Any disagreement was resolved by consensus. Patients were dichotomized into two categories: those with MLS and those without MLS.

The secondary outcomes included the following: (1) parenchymal hematoma type 2 (PH2) on follow-up CT scans, defined as hematoma exceeding 30% of the infarcted volume with significant space-occupying effect (25); (2) 3-month poor outcome, defined by a modified Rankin Scale of 5–6; and (3) 3-month mortality. In addition, for patients with thrombectomy, the successful recanalization rate was assessed on the final run of digital subtraction angiography (DSA), defined as the mTICI score scale of 2b-3.

### Statistical analysis

The statistical analyses were performed using SPSS (version 26; IBM Corporation). The normality of the distribution was analyzed using the Kolmogorov–Smirnov test. Normally distributed continuous data are presented as mean ± standard deviation and assessed using the independent two-sample *t*-test. For data that do not follow a normal distribution, we presented the median along with the interquartile range (IQR) and employed the Mann–Whitney U-test for comparisons. Categorical variables are expressed as percentages and calculated using the chi-square test. The confidence interval was set at 95%, and a *p*-value of < 0.05 was considered statistically significant.

A univariate analysis was performed to assess the prediction of NWU-penumbra and NWU-core in MLS, followed by receiver operating characteristic (ROC) curve analysis. Then, two multivariate regression models, adjusting for patient characteristics, were performed to assess the predictive power of NWU-penumbra and NWU-core in MLS. These showed a statistical significance (*p* < 0.05 for MLS vs. non-MLS groups) or clinical significance. The NWU-core and NWU-penumbra were tested in two models due to the consideration of their collinearity.

A sensitivity analysis was performed on patients with no PH2. A subgroup analysis was executed on patients stratified by collateral circulation status and with or without mechanical thrombectomy.

## Results

### Patients

A total of 133 eligible patients with newly diagnosed anterior circulation infarct who arrived at the hospital within 24 h of symptom onset were enrolled for our final analysis. The baseline characteristics of all the participants are shown in Table 1.

**Table 1:** Patient characteristics and outcomes stratified by MLS.

	**Non-MLS (*n* = 83)**	**MLS (*n* = 50)**	***P*-value**
**Demographics**			
Age, median (IQR)	71 (60.0–79.0)	70 (62.8–79.3)	0.961
Sex, male (%)	50 (60.2)	23 (46.0)	0.110
**Medical history**			
Hypertension, *n* (%)	55 (66.3)	33 (66.0)	0.975
Diabetes mellitus, *n* (%)	6 (7.2)	9 (18.0)	0.057
Atrial fibrillation, *n* (%)	56 (67.5)	26 (52.0)	0.076
Hyperlipemia, *n* (%)	17 (20.5)	13 (26.0)	0.461
NIHSS score on admission, median (IQR)	15 (11–20)	20 (16–26)	**< 0.001** ^*^
**Treatment details**			
Intravenous thrombolysis	41 (49.4)	18 (36.0)	0.132
Mechanical thrombectomy	56 (67.5)	32 (64.0)	0.682
Poor collateral vessel status (score ≤ 2)	53 (63.9)	43 (86.0)	**0.006** ^*^
**Imaging characteristics**			
Ischemic core volume, ml, median (IQR)	22.0 (7.8–36.0)	45.0 (29.0–83.0)	**< 0.001** ^*^
Ischemic penumbra volume, ml, median (IQR)	72.0 (37.0–111.0)	84.0 (58.0–105.0)	0.388
NWU-core, %, median (IQR)	4.9 (2.2–8.1)	6.8 (3.2–10.4)	**0.048** ^*^
NWU-penumbra, %, median (IQR)	0.2 (−2.5–2.7)	2.9 (1.8–4.3)	**< 0.001** ^*^
**Outcomes**			
Recanalization after thrombectomy (TICI grade 2b−3)	47 (84.0)	23 (71.9)	0.178
3-month poor outcome	13 (15.7.0%)	32 (64.0%)	**< 0.001** ^*^
3-month mortality	6 (7.2%)	22 (44.0%)	**< 0.001** ^*^
PH2	0 (0)	9 (18.0)	**< 0.001** ^*^

MLS, midline shift; NIHSS, National Institutes of Health Stroke Scale; NWU-core, net water uptake within ischemic core; NWU-penumbra, net water uptake within ischemic penumbra; PH2, parenchymal hematoma type 2; IQR, interquartile range. The symbol ^*^ means p < 0.05.

Among all the cases, 50 patients (37.6%) were found to have MLS in the follow-up CT scans, whereas 83 did not have MLS (62.4%). Compared with the non-MLS group, patients with MLS had a higher occurrence of poor collateral vessel status [43 (86%) vs. 53 (63.9%), *P* = 0.006], a larger volume of the ischemic core [45.0 (29.0–83.0) vs. 22.0 (7.8–36.0), *p* < 0.001], higher NIHSS scores on admission [22 ± 8 vs. 15 ± 7, *P < * 0.001], a higher NWU within the ischemic core [6.8 (3.2–10.4) vs. 4.9 (2.2–8.1), *P* = 0.048], and a higher NWU within the penumbra [2.9 (1.8–4.3) vs. 0.2 (−2.5–2.7), *P < * 0.001].

Patients who had MLS compared to those with no MLS, patients who had MLS had a higher rate of poor clinical outcome (32 (64%) vs. 13 (15.7%), *p* < 0.001) and a higher rate of 3-month mortality (22 (44%) vs. 6 (7.2%), p < 0.001). For patients with MLS, 9 out of 50 had PH2, whereas no patients had PH2 in the 83 patients without MLS (18.0% vs. 0%, *p* < 0.001). In addition, 88 (66.2%) patients underwent thrombectomy therapy, of whom 70 (79.5%) presented with successful recanalization. However, the rate of successful recanalization after thrombectomy (mTICI grade 2b−3) between the two groups presented no statistical difference (*P* > 0.05).

### NWU predicts MLS in univariate regression

**Figure 2:**
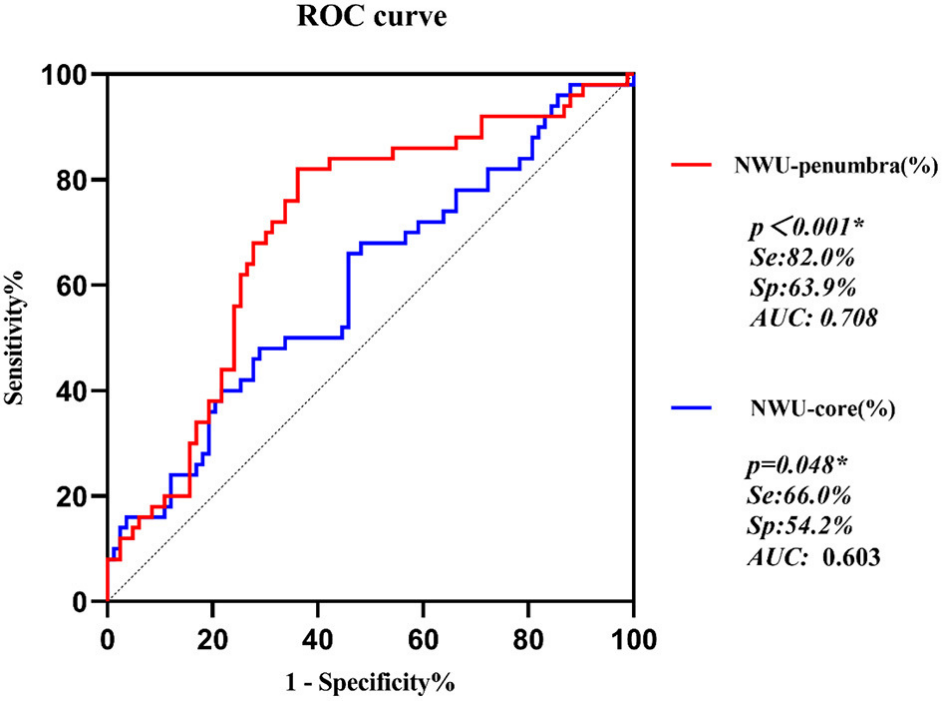
ROC curves of NWU-penumbra and NWU-core on prediction of MLS in acute ischemic stroke patients.

Univariate analyses showed that the NWU either in the core region or the penumbra predicted the presence of MLS (odds ratio = 1.098 (1.012–1.191) and 1.212 (1.079–1.361), respectively, *p* = 0.025 and 0.001, respectively). With an increase in NWU in the core by 1%, the odds of having MLS increased by 9.8%, whereas a 1% increase in NWU in the penumbra resulted in the odds of MLS increasing by 21.2%. ROC analysis showed that NWU-penumbra had significantly higher predictive power than NWU-core in MLS (AUC = 0.708 vs. 0.603, *p* < 0.001, Figure 2).

### NWU predicts MLS in multivariate regression

The results of multivariate logistic regression models are summarized in Table 2. The multivariate regression models included the following predictors: patient characteristics with clinical significance (age, sex, diabetes mellitus, and atrial fibrillation) or statistical significance (NIHSS on admission, poor collateral vessel status, ischemic core volume, NWU-core, and NWU-penumbra; *p* < 0.05 for MLS vs. non-MLS group, Table 1). The NWU-core and NWU-penumbra were tested in two models due to the consideration of their collinearity.

**Table 2:** Multivariate logistic regression analysis for risk of MLS in anterior circulation infarct patients.

**Clinical characteristics**	**All patients**		**Clinical characteristics**	**All patients**	
	**OR (95%CI)**	***P*** **-value**		**OR (95%CI)**	***P*** **-value**
Age	1.007 (0.967–1.049)	0.730	Age	1.014 (0.972–1.058)	0.521
Sex	0.205 (0.061–0.687)	**0.010** ^*^	Sex	0.242 (0.073–0.804)	**0.021** ^*^
Diabetes mellitus	5.160 (1.071–24.865)	**0.041** ^*^	Diabetes mellitus	4.282 (0.831–22.056)	0.082
Atrial fibrillation	0.125 (0.032–0.485)	**0.003** ^*^	Atrial fibrillation	0.127 (0.032-0.508)	**0.004** ^*^
NIHSS score on admission	1.128 (1.038–1.225)	**0.005** ^*^	NIHSS score on admission	1.132 (1.041–1.232)	**0.004** ^*^
Poor collateral vessel status (score ≤ 2)	1.099 (0.324–3.731)	0.879	Poor collateral vessel status (score ≤ 2)	1.006 (0.280–3.618)	0.992
Ischemic core volume	1.033 (1.015–1.052)	**< 0.001** ^*^	Ischemic core volume	1.030 (1.012–1.048)	**0.001** ^*^
NWU-core	1.090 (0.980–1.212)	0.112	NWU-penumbra	1.200 (1.032–1.395)	**0.017** ^*^

MLS, midline shift; NIHSS, National Institutes of Health Stroke Scale; NWU-core, net water uptake within ischemic core; NWU-penumbra, net water uptake within ischemic penumbra. The symbol ^*^ means p < 0.05.

In the multivariate regression model, NWU-penumbra was significantly associated with MLS (adjusted OR = 1.200; 95% CI = 1.032–1.395; *P* = 0.017). However, NWU-core did not show any significance in predicting MLS in the multivariate regression model (adjusted OR = 1.090; 95% CI = 0.980–1.212; *P* = 0.112), adjusting for age, sex, diabetes mellitus, atrial fibrillation, NIHSS on admission, poor collateral vessel status, and ischemic core volume. In both models, higher NIHSS scores and a larger volume of the ischemic core also predicted the presence of MLS, while poor collateral status did not show significant predictive power (Table 2).

### Sensitivity analysis: MLS without large hemorrhages

In this cohort, nine patients had PH2, and all nine patients had MLS. PH2 was significantly associated with MLS (*p* < 0.001). After excluding patients with PH2, the results of the MLS and non-MLS groups are summarized in Table 3. After excluding patients with PH2, NWU-penumbra still showed a significantly higher value in patients with MLS compared to those without MLS [2.9 (1.3–4.3) vs. 0.2 (−2.5–2.7), *P* = 0.001]. In the multivariate regression model (Table 4), NWU-penumbra maintained significant predictive power in MLS (OR = 1.165; 95% CI = 1.002–1.356; *P* = 0.047), adjusting for age, sex, diabetes mellitus, atrial fibrillation, NIHSS on admission, poor collateral vessel status, and ischemic core volume. However, NWU-core had no significant predictive power for MLS in the multivariate regression model (OR = 1.084; 95% CI = 0.967–1.214; *P* = 0.168).

**Table 3:** Characteristics of patients without PH2 stratified by MLS.

	**Non-MLS (*n* = 83)**	**MLS (*n* = 41)**	***P*-value**
**Demographics**			
Age, median (IQR)	71 (60.0–79.0)	70 (61.5–80.0)	0.947
Sex, male (%)	50 (60.2)	21 (51.2)	0.339
**Medical history**			
Hypertension, *n* (%)	55 (66.3)	25 (71.0)	0.562
Diabetes mellitus, *n* (%)	6 (7.2)	5 (12.2)	0.360
Atrial fibrillation, *n* (%)	56 (67.5)	21 (51.2)	0.079
Hyperlipemia, *n* (%)	17 (20.5)	8 (19.5)	0.899
NIHSS score on admission, median (IQR)	15 (11–20)	20 (16.5–27.5)	**< 0.001** ^*^
**Treatment details**			
Intravenous thrombolysis	41 (49.4)	17 (41.5)	0.405
Mechanical thrombectomy	56 (67.5)	24 (58.5)	0.328
Poor collateral vessel status (score ≤ 2)	53 (63.9)	34 (82.9)	**0.029** ^*^
**Imaging characteristics**			
Ischemic core volume, ml, median (IQR)	22.0 (7.8–36.0)	56.0 (33.5–120.5)	**< 0.001** ^*^
Ischemic penumbra volume, ml, median (IQR)	72.0 (37.0–111.0)	84.0 (58.0–103.0)	0.413
NWU-core, %, median (IQR)	4.9 (2.2–8.1)	7.2 (3.2–11.2)	**0.046** ^*^
NWU-penumbra, %, median (IQR)	0.2 (−2.5–2.7)	2.9 (1.3–4.3)	**< 0.001** ^*^
**Outcomes**			
Recanalization after thrombectomy (TICI grade 2b−3)	47 (84.0)	17 (70.8)	0.180
3-month poor outcome	13 (15.7.0%)	24 (58.5%)	**< 0.001** ^*^
3-month mortality	6 (7.2%)	15 (36.6%)	**< 0.001** ^*^

MLS, midline shift; NIHSS, National Institutes of Health Stroke Scale; NWU-core, net water uptake within ischemic core; NWU-penumbra, net water uptake within ischemic penumbra; PH2, parenchymal hematoma type 2; IQR, interquartile range. The symbol ^*^ means p < 0.05.

**Table 4:** Multivariate logistic regression analysis for risk of MLS in anterior circulation infarct patients with no PH2.

**Clinical characteristics**	**All patients with no PH2**		**Clinical characteristics**	**All patients with no PH2**	
	**OR (95% CI)**	***P*** **-value**		**OR (95% CI)**	***P*** **-value**
Age	1.000 (0.959–1.044)	0.982	Age	1.007 (0.964–1.052)	0.749
Sex	0.303 (0.086–1.069)	0.063	Sex	0.316 (0.091–1.099)	0.070
Diabetes mellitus	3.530 (0.584–21.318)	0.169	Diabetes mellitus	3.121 (0.496–19.642)	0.225
Atrial fibrillation	0.174 (0.041–0.737)	**0.018** ^*^	Atrial fibrillation	0.1670.039–0.713)	**0.016** ^*^
NIHSS score on admission	1.122 (1.027–1.227)	**0.011** ^*^	NIHSS score on admission	1.126 (1.030–1.047)	**0.009** ^*^
Poor collateral vessel status (score ≤ 2)	0.856 (0.247–2.962)	0.806	Poor collateral vessel status (score ≤ 2)	0.814 (0.226–2.940)	0.754
Ischemic core volume	1.032 (1.014–1.051)	**< 0.001** ^*^	Ischemic core volume	1.030 (1.012–1.047)	**0.001** ^*^
NWU-core	1.084 (0.967–1.214)	0.168	NWU-penumbra	1.165 (1.002–1.356)	**0.047** ^*^

MLS, midline shift; NIHSS, National Institutes of Health Stroke Scale; NWU-core, net water uptake within ischemic core; NWU-penumbra, net water uptake within ischemic penumbra; PH2, parenchymal hematoma type 2. The symbol ^*^ means p < 0.05.

### Subgroup analysis

For the 124 patients without PH2, subgroup analyses were further conducted. The results of subgroup analyses are summarized in Table 5. Overall, a total of 80 (64.5%) patients experienced mechanical thrombectomy, and 44 patients (35.5%) did not. For patients with mechanical thrombectomy, NWU in the penumbral region predicted MLS (OR = 1.343, *p* = 0.031). However, for patients without mechanical thrombectomy, NWU in the penumbral region had no predictive value in MLS (OR = 1.082, *p* = 0.434).

**Table 5:** Subgroups analysis of NWU-penumbra predicting MLS in patients without PH2.

**Subgroups**	**Non-MLS (*n*)**	**MLS (*n*)**	**Adjusted OR (95%CI)**	***P*-value**	***P* for interaction**
**Mechanical thrombectomy**					**0.002** ^*^
Yes	56	24	1.343 (1.027–1.754)	0.031^*^	
No	27	17	1.082 (0.888–1.319)	0.434	
**Collateral vessel status**					**0.005** ^*^
ASITN/SIR (0–2)	53	34	1.219 (1.024–1.451)	0.026^*^	
ASITN/SIR (3–4)	30	7	1.151 (0.774 to −1.713)	0.487	

MLS, midline shift; NWU-penumbra, net water uptake within ischemic penumbra; PH2, parenchymal hematoma type 2. The symbol ^*^ means p < 0.05.

In addition, 87 patients (65.4%) had poor collateral status, whereas 37 (27.8%) had good collateral status. NWU-penumbra was confirmed as an independent predictor of MLS in those patients with poor collateral status (adjusted OR = 1.219; 95% CI = 1.024–1.451; *P* = 0.026), whereas it did not predict MLS in patients with good collateral status (adjusted OR = 1.151; 95% CI = 0.774–1.731; *P* = 0.487, Table 5).

**Figure 3:**
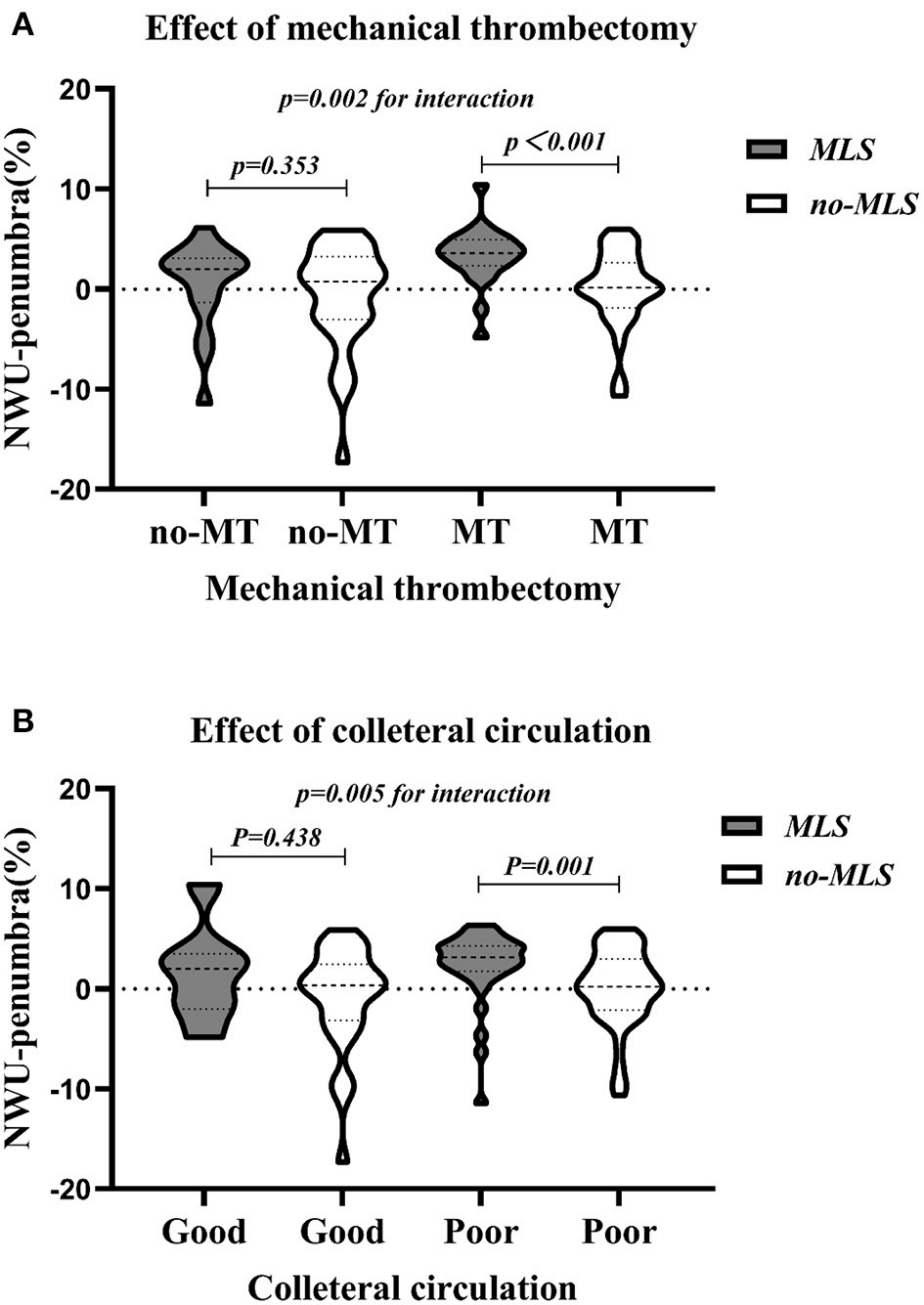
Violin plots showing the dispersion of NWU-penumbra values devised by subgroups. **(A)** With or without mechanical thrombectomy; **(B)** good or poor collateral circulation.

Both mechanical thrombectomy and collateral vessel status had an interaction with NWU-penumbra in predicting MLS (*p* = 002 and *p* = 0.005, respectively). Figure 3A demonstrates that the value of NWU-penumbra was only statistically different between the MLS and non-MLS groups when patients had mechanical thrombectomy, while Figure 3B indicates that the value of NWU-penumbra was only statistically different between the MLS and non-MLS groups when patients had poor collateral statuses.

## Discussion

The main finding of this study is that increased net water uptake within the acute penumbral region was significantly associated with severe brain edema at 24–48 h, measured by MLS on the brain CT. When excluding patients whose MLS was caused by a large hemorrhagic transformation, a significant association still existed between penumbral NWU and MLS.

To the best of our knowledge, this is the first study that reports the relationship between net water uptake within the ischemic penumbra and follow-up severe brain edema. A previous study from our group reported a positive relationship between penumbral net water uptake and hemorrhagic transformation on follow-up images (20). A high reperfusion/recanalization rate has been reported in both studies. Together, the two studies indicate a potential relationship between penumbral NWU and reperfusion injuries. Although the penumbra has been the target of reperfusion treatment, in patients with high NWU of the penumbral region, such treatment might cause reperfusion injuries of edema or hemorrhagic transformation. In this study, for patients with mechanical thrombectomy, NWU-penumbral in the MLS group was significantly higher than in the non-MLS group. In addition, NWU in the penumbral region predicted MLS, but not for patients without mechanical thrombectomy. The findings of this study suggest that the optimal candidate for reperfusion treatment would be patients with penumbral tissues to be salvaged but also with a limited increase of NWU within the penumbral region, which could potentially lead to the maximum benefit and minimum risk from the treatment.

Another novel finding of the study is the interaction of penumbral NWU and collateral vessels in predicting severe brain edema. The association between penumbral NWU and MLS was found to be limited to patients with poor collateral vessel status. One possible explanation is that the penumbral NWU reflects how much edema can expand, and the edema expansion also depends on the collateral status. Such an assumption is supported by the recent studies of collateral status and NWU. Broocks et al. (26) reported that patients with poor collateral status had significantly higher early edema progression rates, while Galego et al. (27) reported that poor collateral circulation was independently associated with significant cerebral edema 24 h after acute ischemic stroke in patients who underwent reperfusion treatment.

This study also found an increased net water uptake in the ischemic core in patients with MLS. However, the core NWU did not predict MLS in the multivariate regression model when adjusting for confounders, including baseline NIHSS and acute core volume. This was different from the findings of the previous studies reporting that the NWU within the ischemic core predicted malignant brain edema (8, 15, 16). One possible explanation is the different definitions of NWU in previous studies. Both image patch-based NWU and ASPECTS-NWU were dependent on NECT rather than CTP and possibly failed to differentiate the penumbra from the ischemic core (8, 15). Another explanation did not account for the effect of parenchymatous hemorrhage on malignant brain edema (16). Parenchymatous hemorrhage with a space-occupying effect may contribute to the overstatement of the predictive ability of NWU-core for malignant brain edema in previous studies. Finally, the CTP-derived parameters have been criticized recently for overestimating the final infarct core (28, 29). Accordingly, the MLS prediction ability of NWU-core, which CTP recognizes, may similarly be biased.

This study has several limitations. First, this is a retrospective and single-center study, which may result in a selection bias of patients with and without MLS. Multivariate regression was performed to adjust for such bias. However, residual confounders might exist. The findings of this study need to be validated in a prospective study and, ideally, with a larger sample size recruited from multiple centers. Second, due to the small sample size, this study did not further grade MLS according to different degrees of horizontal displacement. In future studies, graded MLS can help further explore the relationship between brain edema and net water uptake. Finally, the findings of this study are limited to patients with anterior circulation strokes. The accurate definition of core and penumbra is limited to anterior circulation stroke, whereas limited data are available on how to delineate core and penumbra in posterior circulation stroke. Most NWU studies, including this study, are limited to patients with anterior circulation stroke.

## Conclusion

In conclusion, it is important to measure net water uptake in the penumbral region acutely since it can potentially predict severe brain edema, especially in patients with poor collateral status.

## Data availability statement

The original contributions presented in the study are included in the article/supplementary material, further inquiries can be directed to the corresponding authors.

## Ethics statement

The studies involving human participants were reviewed and approved by the First Affiliated Hospital of Ningbo University. The patients/participants provided their written informed consent to participate in this study.

## Author contributions

CC: data curation, statistical analysis, and organizing and writing the original draft. JY: project administration. QH, YW, JL, and TX: investigation and resources. JS, XG, and YH: funding acquisition. MP and LL: validation and writing—review and editing. All authors contributed to the article and approved the submitted version.
